# Crossing species boundaries in regenerative neuroscience with rat–mouse brain chimeras

**DOI:** 10.1038/s41684-024-01394-3

**Published:** 2024-06-17

**Authors:** Stefano Pluchino, Ivan Lombardi

**Affiliations:** 1 Department of Clinical Neurosciences and NIHR Biomedical Research Centre, University of Cambridge, Cambridge, UK; 2 Department of Biotechnology and Biosciences, University of Milano-Bicocca, Piazza della Scienza, Milan, Italy

**Keywords:** Neuroscience, Biological techniques

## Abstract

Understanding the inherent complexity of organogenesis and addressing the persistent shortage of organ donors remain paramount scientific challenges. Recent advances in chimeric blastocyst technology offer promising solutions. Two new pioneering studies have successfully generated functional rat–mouse brain chimeras, providing novel insights into brain development and potential regenerative therapies. However, several technical and ethical hurdles persist.

The shortage of organ donors, alongside the ongoing challenge of comprehensively elucidating the intricate cellular and molecular processes underlying organ development, still represents unmet scientific needs^1^. Nonetheless, technical advances in the generation of newly designed chimeras provide a unique tool that can help address these gaps. Interspecies blastocyst complementation (IBC), based on modified blastocyst chimeras with a specific empty cellular niche, has been successfully implemented over the past years for the generation of different organ chimera models, such as of pancreas^2^ or kidney^3^. Although the generation of hybrid brains (e.g., healthy or diseased human neurons/organoids transplanted in rodent brains) has already been reported^4,5^, so far, the generation of fully functional brain tissue via IBC has not yet been achieved.

Scientists at independent research institutions have now described an improved blastocyst complementation method that allowed for the first time the generation of rat–mouse functional forebrain chimeras.

Huang and colleagues^6^ pioneered an optimized precise gene-editing technology (named C-CRISPR-based blastocyst complementation, CCBC) to pinpoint genes crucial for tissue-specific development. Through this innovative approach, they identified *Hesx1* as a critical gene for normal murine forebrain formation. The deletion of *Hesx1* from mouse blastocysts resulted in the demise of the animals shortly after birth. However, injecting lab-grown rat-derived embryonic stem cells (rESCs) into murine *Hesx1* knock-out (*Hesx1*^–/–^) blastocysts led to the generation of rat–mouse chimeras with partial (in 401/417 animals) or full (in 16/417 animals) forebrain reconstitution (Fig. 1). Remarkably, these chimeras exhibited normal growth and behaviour, indicating the seamless integration and functional compatibility of rat-derived cells within the host organism. Further analysis unveiled that the development of rat-derived brain tissue was governed by non-cell autonomous mechanisms, mirroring the pace of host tissue development. These findings contrast with previous xenotransplantation studies, where transplanted cells typically retained species-specific features despite maturing in accelerated hosts^7^. For instance, human cells injected into the central nervous system of mice or rats maintained their human-specific slower proliferation and differentiation rate, underscoring the challenge of prolonged experimental time points in stem cell transplantation studies^8^. Nonetheless, the transcriptomic signature of rat-derived cells was found to be regulated by an inner cell-autonomous mechanism, with rESCs-derived neurons closely resembling those of wild-type rats^6^.

**Fig. 1:**
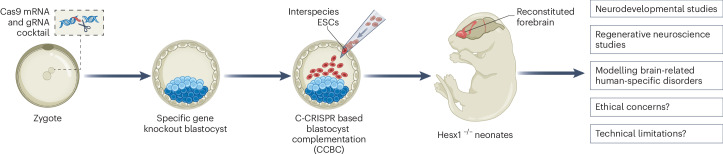
The promise of interspecies blastocyst complementation (IBC) technologies in regenerative neuroscience. Recent advances in precise gene-editing technology have enabled the generation of blastocysts with specific empty cellular niches. The *Hesx1* knock-out mouse model (*Hesx1*^–/–^), which results in complete forebrain ablation, combined with interspecies embryonic stem cell (ESC) transplantation, offers a promising avenue for future neurodevelopmental, disease modelling and regenerative neuroscience studies. However, ethical concerns and technical limitations persist.

Throesch et al.^9^ pursued a different yet complementary approach by investigating the potential of rat-derived ESCs to integrate, develop, differentiate and restore disrupted neural sensory circuits in two anosmic mouse models. These in vivo murine models were created by genetically disabling or eliminating their olfactory sensory neurons (OSNs), rendering them unable to use their sense of smell to locate food. When rat stem cells were introduced into the blastocysts of these mice, a partial recovery was observed only in the latter model, while the ‘silence’ models did not exhibit successful restoration of food-seeking behaviour. The authors hypothesized that this discrepancy may stem from incomplete contribution of rat cells to murine brains, coupled with unknown interspecific differences governing synaptic connectivity and behaviour, accumulated over 10–20 million years of evolutionary divergence. Nevertheless, these findings underscore the partial plasticity of synthetic brain cells in innervating and promoting limited recovery of damaged or absent neuronal structures.

Taken together, these two studies offer a dual contribution. Firstly, they provide a technical framework to address ongoing inquiries into the intricate development of the central nervous system and the global shortage of organs. Secondly, they offer valuable insights for regenerative neuroscience, particularly in challenging the hypothesis that synthetic neural circuits from different species can effectively restore brain functions lost during aging or neurodegeneration. However, despite their biological intrigue and methodological novelty, both studies face substantial limitations that warrant consideration.

A notable main hurdle arises in the efficiency of generating fully complemented rat–mouse brain chimeras. In fact, only 16/417 mice (3.84%) exhibited a complete rat-derived forebrain. An even lower survival rate was observed in attempts to accurately differentiate between rat and mouse neurons; co-injecting Cas9 mRNA and *Hesx1* gRNAs into mouse zygotes expressing EGFP, followed by the injection of tdTomato-labelled rESCs into 956 blastocysts, yielded only eight (0.83%) post-natal chimeras co-expressing EGFP and tdTomato, with merely two of them persisting beyond postnatal day 14 (25%)^6^.

Another notable challenge is the potential presence of a xenogeneic barrier, which strikingly diminishes the rate of chimeric rESCs during mid-to-late mouse developmental stages (from 100% to ~60% in the forebrain by E15.5)^6^. Furthermore, the broader application of these chimeras for neurodevelopmental or organ/tissue regenerative studies is hindered by the stochastic distribution of donor cells across various brain regions among different animals. This inherent heterogeneity makes it exceedingly difficult to fully adhere to the 3R principles, as Throesch et al.^9^ reported that they had to generate over ~900 rat–mouse chimeras over a span of four years to achieve a sufficiently robust statistical power for their behavioural assessments.

As the quest to generate fully functional, transplantable human organs in large animals progresses, ethical considerations and heightened attention surround its application in neuroscience. Central to the debate is the prospect of creating animals with markedly human-like brains through advanced technologies. Given that neural networks shape fundamental aspects of human cognition and identity, ethical use of this technology should prioritize investigations into neurodevelopment and complex, human-specific neural disorders^10^. These conditions, often challenging to study in vitro due to intricate cellular interactions, warrant focused exploration without necessitating the creation of complete human-like brains in animal models for transplantation purposes.

In light of these considerations, it becomes evident that blastocyst complementation remains a distant prospect for human clinical applications. Nevertheless, it continues to serve as a captivating tool for deepening our understanding of brain disorders, particularly those related to neurodevelopment. Moreover, it offers a promising avenue for developing effective strategies to address these disorders. Further research aimed at overcoming associated limitations and addressing ethical concerns will undoubtedly shed light on the broader potential applications of this powerful technology.
